# Safety evaluation of a novel variant of consensus bacterial phytase

**DOI:** 10.1016/j.toxrep.2020.07.004

**Published:** 2020-07-15

**Authors:** Gregory S. Ladics, Kang-Hyun Han, Min S. Jang, Heejin Park, Valerie Marshall, Yueming Dersjant-Li, Vincent J. Sewalt

**Affiliations:** a DuPont Nutrition and Biosciences, 200 Powder Mill Road, Wilmington, DE, USA; b Department of Advanced Toxicology Research, Korea Institute of Toxicology, 141 Gajeong-ro, Yuseong-gu, Daejeon, 34114, Republic of Korea; c DuPont Nutrition and Biosciences, 1501 Larkin Center Drive, Midland, Michigan, USA; d DuPont Animal Nutrition, DuPont Nutrition and Biosciences, Archimedesweg, 30, 2333 CN, Leiden, the Netherlands; e DuPont Nutrition and Biosciences, 925 Page Mill Road, Palo Alto, CA, 94304, USA

**Keywords:** bw, body weight, BLAST, basic local alignment search tool, DM, dry matter, FTU, phytase units, IP6, myo-inositol hexaphosphate, NOAEL, no-observed-adverse-effect-level, MCP, monocalcium phosphate, OECD, The Organisation for Economic Co-operation and Development, SSL, safe strain lineage, TOS, total organic solids, UFC, ultra-filtered concentrate, Phytase, NOAEL, Genetic toxicology, Subchronic study, Oral gavage

## Abstract

A 90-day subchronic oral toxicity study was conducted to evaluate the safety of a consensus bacterial phytase variant 6-phytase (PhyG) for use as an animal feed additive. This phytase is produced by fermentation with a fungal (*Trichoderma reesei*) production strain expressing a biosynthetic variant of a consensus bacterial phytase gene assembled *via* ancestral reconstruction with sequence bias for the phytase from *Buttiauxella sp*. Rats were administered PhyG daily *via* oral gavage at dose-levels of 0 (distilled water), 250, 500 or 1000 mg total organic solids (TOS)/kg bodyweight (bw)/day (equivalent to 0, 112,500, 225,000 and 450,000 phytase units (FTU)/kg bw/day, respectively). No test article-related adverse effects were observed. A no-observed-adverse-effect level (NOAEL) for PhyG was established as 1000 mg TOS/kg bw/day, the highest test concentration. Based on this NOAEL and an estimate of broiler consumption determined from the proposed inclusion of the phytase in feed at the maximum recommended level (4000 FTU/kg), a margin of safety value of 1613 was calculated. Results of *in vitro* genotoxicity testing and *in silico* protein toxin evaluation further confirmed PhyG to be non-genotoxic and not likely to be a protein toxin upon consumption. These data support the safety of PhyG as an animal feed additive.

## Highlights

• 90-day oral and genetic toxicology studies were conducted on a next generation bacterial biosynthetic 6-phytase as an animal feed additive.
• No test article-related adverse effects were observed and a NOAEL was established as 1000 mg Total Organic Solids/kg bw/day.
• A margin of safety value of 1613 was calculated based on the NOAEL and an estimate of broiler feed consumption.
• Data support the safety of PhyG as an animal feed additive.

## 1 Introduction

Pigs and poultry diets are typically based on cereals and oilseeds in which up to 70–80 % of the phosphorus (P) content is bound to phytate (the salt of phytic acid, myo-inositol hexaphosphate; IP6) [1]. Phytate is poorly utilized by monogastric animals (pigs, poultry) due to a lack of endogenous phytase activity. Phytase (myo-inositol hexakisphosphate phosphohydrolase) is commonly added to commercial pig and poultry diets to improving the bioavailability of P from phytate. The exogenous phytase not only increases the availability of P to the animal [[2], [3], [4]] but can also improve digestibility of other key nutrients [[5], [6], [7]]. Further, the inclusion of phytase in monogastric diets reduces the need to add costly inorganic sources of phosphorus and reduces P excretion [8].

The bioefficacy of supplemental phytase in pig and poultry diets is well established [1,9,10]. Since the first generation of microbial phytase commercialized in the 1990s, new generation phytases have been developed [11]. These new phytases have increased bioefficacy and may also possess other desirable characteristics such as more active in upper gastro-intestinal tract to achieve rapid and complete phytate hydrolysis, increased substrate affinity/specificity and/or enhanced thermal stability to better withstand the high temperatures associated with the pelleting process. For example, a current generation 6-phytase derived from *Buttiauxella* sp. and dosed at 1000 FTU/kg can achieve 76–92 % ileal phytate degradation in broilers [[12], [13], [14]] and 76 % ileal phytate degradation in pigs [15]. Nevertheless, new phytases with further enhanced activity and more desirable biochemical and thermal properties continue to be sought.

The phytase enzyme described in this paper, herein described as ‘PhyG’, is a consensus bacterial 6-phytase variant (EC 3.1.3.26) that was recently demonstrated to have high *in vivo* efficacy in both broiler and pig diets [16,17].

Oral toxicity testing using rodent models is a well-established approach for evaluating the safety of dietary additives to support their safe use in food and feed applications, and such testing is typically performed according to guidelines set by the Organisation for Economic Co-operation and Development (OECD). Recent examples of the successful use of oral toxicity testing approaches as part of safety evaluations of dietary additives include a subchronic (90 day) toxicity test of fibrillated cellulose in rats [18] and an acute oral toxicity evaluation of three amino acid additives containing *Corynebacterium Glutamicum* biomass [19]. The study by Ong et al. [18] utilized a similar study design and was performed according to the same OECD Test Guideline as the present study.

The aim of the present study was to evaluate the safety of the consensus bacterial phytase variant PhyG for use as a feed additive in animal feed by performing a 90-day repeated dose subchronic oral toxicity study in rats [20] and *in vitro* genetic toxicology studies [Ames assay [21] and Chromosomal Aberration [22] assays]. In addition, the similarity of PhyG to protein toxins was evaluated using in silico procedures [23].

## 2 Materials and methods

### 2.1 Phytase preparation

The experimental phytase, PhyG, was developed and manufactured by DuPont Nutrition and Biosciences (Wilmington, DE, USA) as described below. This variant was developed *via* ancestral reconstruction based on available bacterial phytase sequences, with sequence bias for *Buttiauxella* sp phytase. This resulted in a consensus biosynthetic bacterial phytase sequence with high- melting temperature, which was subjected to further protein engineering with multiple amino acid substitutions and screening for improved enzymatic performance. Phytase variants were produced in a fungal expression system using the filamentous fungus *Trichoderma reesei* as a production organism. A synthetic, codon optimized gene encoding PhyG phytase under the control of the strong cbhI promoter was integrated into the fungal genome and transformants were screened for phytase activity. After the fermentation process, followed by removal of the production strain and cell debris using depth filtration with filter aids, the purified enzyme in liquid form was concentrated and desalted using ultrafiltration with a MWCO of 10,000 Daltons to produce the test article which was provided as a frozen (-20 °C) clear brown liquid. The same manufacturing lot was used in all studies. The test article met the established product specifications based on analytical testing and was determined as free from microbial and heavy metal contamination. The UFC (ultra-filtered concentrate) test article contained 206.47 mg/g total protein, a specific gravity of 1.063 g/ml and a total organic solids (TOS) content of 21.75 % (analyzed by Silliker Laboratories (Texas, USA)], with an activity of 97,885 phytase units (FTU) per gram of test material, equivalent to 450 FTU per mg TOS or 474 FTU per mg total protein (where 1 mg TOS =0.95 mg total protein). One phytase unit (FTU) was defined as the amount of enzyme that released 1 μmol of inorganic orthophosphate from a sodium phytate substrate per minute at pH 5.5 and 37 °C [24]. The test article was stored in an airtight container under frozen conditions (-20 °C). Analytical testing conducted by the manufacturer indicated that the product was enzymatically stable throughout the duration of the study.

### 2.2 90-day oral toxicity study

This study was conducted at the Korea Institute of Toxicology, a facility that has received accreditation by the Association for Assessment and Accreditation of Laboratory Animal Care International (AAALAC International). All animal care and use activities required for the conduct of the study were reviewed and approved by the Institutional Animal Care and Use Committee of the test facility and carried out in accordance with the Animal Welfare Act and Guide for the Care and Use of Laboratory Animals (Institute for Laboratory Animal Research, ILAR).

#### 2.2.1 Diets

During acclimation and throughout the experimental period, animals were provided *ad libitum* with a standard commercial irradiated pelleted chow diet (Lab Diet #5053, PMI Nutrition, USA). Drinking water was obtained from municipal tap water, filtered and ultraviolet light-irradiated and provided *ad libitum* throughout the duration of the study.

#### 2.2.2 Preparation and analysis of dosing formulations

Three dose-levels of the test article were prepared by diluting the thawed stock solution in 0%, 25 % or 50 % distilled water. These dose-levels were tested against 100 % distilled water as the control vehicle. The respective dilutions resulted in a daily dosage of the test article of 0 (distilled water), 250, 500 and 1000 mg TOS/kg bw/day, based on the analyzed TOS content of the test article of 21.75 %. These dose levels were equivalent, respectively, to 112,500, 225,000 and 450,000 FTU/kg bw/day, based on the known activity and percentage TOS of the product described in section 2.1. The test dose-levels were selected based on the OECD limit dose of 1000 mg/kg/day (and dilutions thereof) on the basis that enzymes are not considered toxic by the oral route [25,26]. Dosing formulations were prepared and used within the analytically established range of stability of the test article and refrigerated at -4 °C before use. Dose formulations were collected on three occasions during the experiment (beginning, middle, end), frozen, and analyzed together for concentration of protein (mg/mL) by CHNS Elemental analysis using a nitrogen/protein analyzer (Fisons EA 1108 CHNS-O Element Analyzer, Thermo Fisher Scientific, USA) to verify target dose levels.

#### 2.2.3 Animals and treatment

A total of 88 (44 male:44 female) five-week-old Crl:CD Sprague-Dawley rats were obtained from Orient Bio Inc. (Gyeonggi-do 13201, Republic of Korea) and acclimatized for 5 days in the laboratory. Following acclimatization, 40 animals of each sex that were in good health and of approximately equal body weight (227.5–259.0 g in males and 156.7–198.2 g in females) were randomized into one of four treatment groups so that each group contained equal numbers of males and females, using the Pristima software system (Xybion, NJ, USA). Animals were housed in solid bottomed stainless-steel cages (≤2 same-sex animals per cage) for the duration of the 90-day experimental period. Cages were housed in an animal room maintained at 22 ± 3 °C, 30–70 % relative humidity and a 12 h light-dark cycle, with ventilation 10–20 times/h. Heat-treated laboratory wood chip (Tapvei Estonia OÜ, Estonia) was used for bedding. Following a pre-treatment period of 3 days for males and 4 days for females, animals were administered by oral gavage with a daily dose of vehicle or PhyG for 90 days. The daily doses were administered at a volume of 4.33 mL/kg bw. Dosing took place at approximately the same time each day (±3.5 h).

#### 2.2.4 Clinical observations, body weights, feed consumption and ophthalmology

All animals were observed twice daily for mortality, morbidity and behavior changes, except on the day of necropsy (day 91) when observations were made once. Animals were weighed individually at the start of treatment and weekly thereafter, including on the day of necropsy. Feed consumption per cage was measured once at the end of the pre-treatment phase and weekly thereafter and used to calculate the mean feed consumption per animal per week. Ophthalmological examinations were conducted once prior to randomization and during the final week of the experiment by a veterinary ophthalmologist using a binocular indirect ophthalmoscope (IO-H Neitz Instrument Co., Japan, or Vantage Plus Digital, Keeler Ltd., England), after treatment of animals with a mydriatic solution (Mydrin-P, Santen Pharmaceutical Co., Japan).

#### 2.2.5 Neurobehavioral evaluation

An abbreviated neurobehavioral test battery, consisting of selected functional observation battery assessments (FOB) and motor activity sessions (MA), was conducted on all study animals during the pretreatment period and again during the final week of treatment (day 86–90). The FOB assessments included standard home cage and outside the home cage observations, open field arena observations, manipulations assessment, grip strength, landing foot splay and body temperature. Motor activity sessions evaluated the amount of spontaneous movement during 60 min using Etho Vision XT Version 8.5 (Noldus International Technology B.V., Netherlands).

#### 2.2.6 Hematology and coagulation

Animals were fasted for 16 h prior to blood sample collection. Blood (∼1.5 mL) was collected from the abdominal vena cava of all animals at terminal sacrifice whilst under deep isoflurane anesthesia. Blood samples were mixed with potassium salt of EDTA and assayed for parameters listed in Table 3, using an ADVIA2120i hematology analyzer (Siemens, USA). Clotting potential was assessed by mixing ∼1.0 mL blood with 3.2 % sodium citrate solution prior to centrifugation at ∼3000 rpm for 10 min at room temperature. Plasma was collected and analyzed for prothrombin time using an ACL 9000 coagulation analyzer (Instrumentation Laboratory, Italy).

#### 2.2.7 Clinical chemistry

Blood samples (∼1.5 mL) were collected as described in section 2.2.6 and serum separated by mixing with anticoagulant. Tubes were maintained at room temperature for at least 90 min and then centrifuged (∼ 3000 rpm, 10 min, room temperature) to obtain serum. Serum parameters were analyzed using a Toshiba 200FR NEO Chemistry Analyzer (Toshiba Co., Japan).

#### 2.2.8 Urinalysis

Urine was collected overnight (∼ 16 h period) the day before terminal sacrifice. Animals were housed in individual metabolic cages during this period. Water was available *ad libitum* but animals were fasted. Urine volume was measured and the following parameters were analyzed using a Cobas U411 uring analyzer (Roche, Germany) and Combur 10 T M urine sticks (Roche, Germany): pH, specific gravity, bilirubin, protein, urobilinogen, nitrite, glucose, erythrocytes and ketones. Urine cast, epithelial cells, white blood cells and red blood cells were examined by microscopy of urine micro sediments.

#### 2.2.9 Hormone analysis

Blood samples (∼ 0.7 mL) were collected from all animals as described in section 2.2.6 and serum obtained as described in section 2.2.7. Collected serum was stored frozen until analysis. Hormones were analyzed using in-house validated methods. Thyroid stimulating hormone was measured using a SpectraMax 190 or VersaMax UV Microplate Reader (Molecular Devices, USA) and Tri-iodothyronine and thyroxine were analyzed using an API 5000 LC/MS/MS system (Applied Biosystems, USA).

#### 2.2.10 Organ weights, Gross pathology and histology

All animals were euthanized on day 91 by exsanguination under isoflurane anesthesia following blood sampling. All animals underwent full pathological examination, abdominal, thoracic and cranial cavities were examined for abnormalities and the organs were removed, examined and weighed. Both absolute and relative-to-body weight (as measured prior to terminal sacrifice) organ weights were determined. For histopathology, bone marrow smears were prepared using bone marrow collected from the left femur and vaginal smears were prepared from all female animals. The eyes (with optic nerve) were fixed in Davidson’s fixative and the testes and epididymides were fixed in Bouin’s fixative for 48 h prior to transfer to 70 % ethanol. Formalin was infused into lungs (*via* the trachea) and the urinary bladder. Other tissues and organs as specified OECD test guideline 408 [20] were preserved in 10 % neutral buffered formalin. All tissues collected at sacrifice from all treatments were stained with hematoxylin and eosin and examined microscopically for abnormalities.

### 2.3 Genotoxicity studies

The following genotoxicity studies were carried out by BioReliance Corporation, Rockville, MD, USA.

#### 2.3.1 Bacterial reverse mutation test (Ames test)

The mutagenicity of the test article in *Salmonella typhimurium* and *Escherichia coli* was evaluated by performing the Ames test, in accordance with OECD Test Guideline No. 471 [21]. Briefly, the study was conducted in *Salmonella typhimurium* strains TA98, TA100, TA1535 and TA1537 and in *E. coli* strain WP2 uvrA.

The assay was performed in two phases using the treat and plate method (a modification of the preincubation method) [27], except for the test involving the positive control for *E. coli* in the presence of metabolic activation, in which the plate incorporation method was used [28]. The first phase consisted of an initial toxicity-mutation assay to establish the appropriate dose-range for the mutagenicity assay. The second phase constituted the confirmatory mutagenicity assay itself.

Aroclor 1254-induced rat liver S9 was used as the metabolic activation system and purchased from Moltox (Boone, NC, USA). Distilled water was used as the diluent for the test article (PhyG). Positive controls in the presence of S9 activation comprised 2-aminoanthracene (Sigma Aldrich) and in the absence of S9 activation, comprised: 2-nitrofluorene (Sigma Aldrich), N-methyl*-N-*nitro*-N-*nitrosoguanidine (MNNG; TCI America) and ICR-191 (Sigma Aldrich). All positive controls were diluted with dimethyl sulfoxide (DMSO). For each replicate plating, the mean and standard deviation of the number of revertants per plate were calculated and are reported.

Based on laboratory historical control data (and derived 95 % control limits), all tester strain cultures were required to exhibit characteristic numbers of spontaneous revertant colonies per plate with the vehicle controls that fell within the established 95 % control limits (inclusive) set out in Table 1, for the assay to be considered valid.

**Table 1:** Control limits (95 %) for the interpretation of data (no. or revertant colonies per plate) from tester strains and untreated controls in the Bacterial Reverse Mutation Test (Ames Test). Control limits were inclusive and based on historical control data.

	95 % Control limits (99 % Upper Limit)^2^ for no. of spontaneous revertant colonies/plate, applied to bacterial tester strains				
Presence/absence of metabolic activation^1^	TA98	TA100	TA1535	TA1537	WP2 *uvr*A
-S9 (without metabolic activation)	5−25 (30)	66−114 (126)	4−20 (24)	2−14 (17)	10−38 (45)
+S9 (with metabolic activation)	10−34 (40)	66−122 (136)	4−20 (24)	3−15 (18)	13−41 (48)

1 Aroclor 1254-induced rat liver S9 was used as the metabolic activation system.

2 Calculated from laboratory historical control data, where: 95 % upper control limit = mean + (2 x standard deviation); 95 % lower control limit = mean – (2 x standard deviation); 99 % upper control limit = mean + (3 x standard deviation). With Study Director justification, values including the 99 % upper control limit and above were considered acceptable.

The mean of each positive control was required to exhibit at least a 3.0-fold increase in the number of revertants over the mean value of the respective vehicle control. A minimum of three non-toxic dose levels was required to evaluate assay data. A dose level was considered toxic if one or both of the following criteria were met: (a) A > 50 % reduction in the mean number of revertants per plate as compared to the mean vehicle control value, accompanied by an abrupt dose-dependent drop in the revertant count. (b) At least a moderate reduction in the background lawn (background lawn code 3, 4, or 5).

#### 2.3.2 *In vitro* mammalian chromosomal aberration test

The *in vitro* mammalian chromosome aberration test was performed in accordance with OECD Test Guideline No. 473 [22]. The test was conducted in human peripheral blood lymphocytes (HPBL) obtained from two healthy non-smoking males. This system has been demonstrated to be sensitive to the clastogenic activity of a variety of chemicals [29].

Araclor 1254-induced rat liver S9 was used as the metabolic activation system and was purchased from Moltox (Moltox, Boone, NC, USA). Water (Gibco, Thermo Fisher Scientific) was used as the vehicle to deliver the test article, PhyG, to the test system. Positive controls included Mitomycin C (MMC, Sigma-Aldrich) in the non-active test system and Cyclophosphamide (CP, MP Biomedicals) in the S9-activated system. The mitotic index was recorded as the percentage of cells in mitosis per 500 cells counted. Metaphase cells were examined under oil immersion without prior knowledge of treatment groups (300 metaphases/dose level; 150/duplicate treatment) for chromatid-type and chromosome-type aberrations including chromosome/chromatid breaks, exchanges and other aberrations [30]. In addition, the percentage of cells with numerical aberrations (polyploid and endo reduplicated cells) was evaluated for 150 cells per culture (300/dose level). A positive response was assigned if 1) the incidence of aberrant cells in at least one of the test concentrations was significantly higher than the concurrent negative control, 2) the increase was concentration related, and 3) the results were outside the 95 % control limit of the historical negative control data.

### 2.4 Similarity to protein toxins

*In silico* bioinformatics analysis of the amino acid sequence of PhyG expressed in *T. reesei* was conducted to compare sequence homology against known toxins and venoms. A basic local alignment search tool (BLAST) search for homology of the phytase sequence with proteins listed in the complete Uniprot database (http://www.uniprot.org) was performed on 10th October 2018. The UniProt annotated protein knowledge database release 2018_09 of 10th October 2018 obtained 558,590 reviewed proteins of which 5962 sequences were manually annotated as toxins and 6406 as venom proteins. An additional more specific BLAST search was performed for homology with proteins in the Uniprot animal toxin database. Bioinformatic analyses used a threshold *E*-value of 0.1.

### 2.5 Statistical analysis

For the 90-day study, data are reported by individual animal as the experimental unit, except for feed consumption which was determined by cage and used to calculate the mean consumption (g) per animal per week. Bartlett’s test was applied to test for the homogeneity of variance across treatment groups, for all measured parameters. Homogeneous data were analyzed using Analysis of Variance (ANOVA) followed by Dunnett’s test to identify significant differences between active treatments and the control group. Heterogeneous data were analyzed by the Kruskall-Wallis test followed by Dunn’s rank sum test to identify differences between active and control treatments. Statistical analysis of neurobehavioral data was conducted in SAS (version 9.4). All other statistical analyses were conducted in Pristima (version 7.4). For the chromosomal aberration study, statistical analysis was performed using the Fisher's exact test (p ≤ 0.05) for a pairwise comparison of the frequency of aberrant cells in each treatment group with that of the vehicle control. The Cochran-Armitage trend test was used to assess dose-responsiveness.

## 3 Results

Analyzed dose formulations (mg total protein per ml) were within ±11.1 % of target dose levels in all cases (data not shown).

### 3.1 90-day oral toxicity study

#### 3.1.1 Mortality

One male rat (250 mg TOS/kg bw/day treatment group) was found dead on day 84. Pathology revealed no treatment-related changes in macroscopic or microscopic observations. The cause of death was not determined but considered incidental and not test article-related. All other animals survived until the scheduled sacrifice on day 91 of the experiment.

#### 3.1.2 Clinical and cage-side observations

No treatment-related clinical signs were observed in any animals in any treatment group. Loss of fur was observed at low frequency and sporadically across treatment groups. This was not considered to be test article-related.

#### 3.1.3 Ophthalmology

Slight opacity of the left eye was observed in one female rat (1000 mg TOS/kg bw/day treatment group). There were no correlated changes in macroscopic or microscopic examinations, thus it was considered unrelated to the test article. No ophthalmological abnormalities were observed in any other animals in any treatment group.

#### 3.1.4 Body weight, body weight gain, feed consumption

**Fig. 1:**
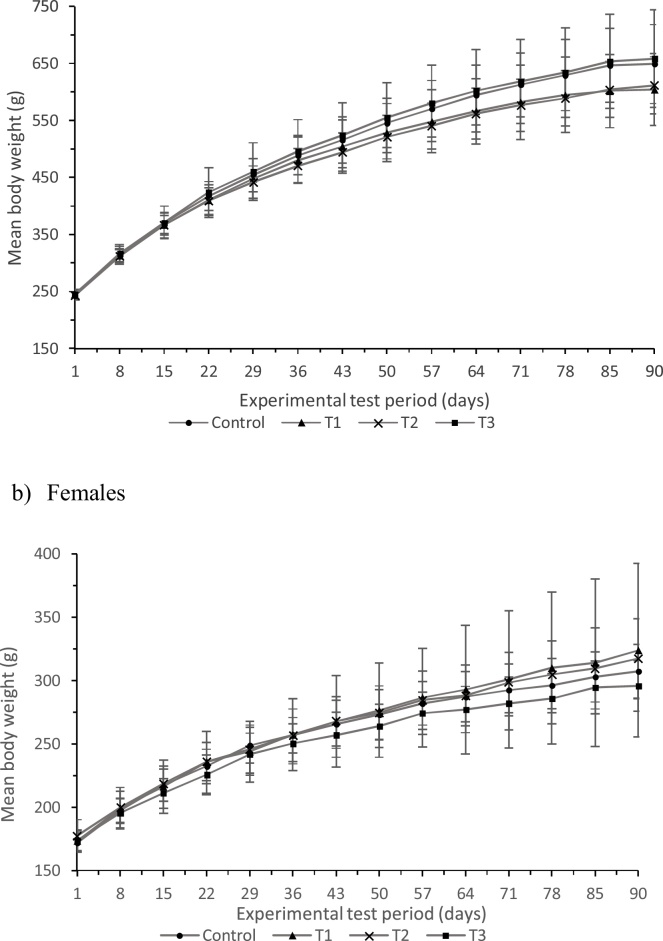
Mean weekly body weights of a) male and b) female rats administered a daily dose of PhyG by oral gavage for 90 days. No statistically significant differences were detected. Error bars are standard deviations of the mean value.

No differences were identified in the mean body weights of female or male rats given the experimental phytase at any of the three dose levels, at any time-point, when compared with their respective controls (Fig. 1). Body weight gains of all treatment groups were also comparable with the respective controls in both male and female rats at all time-points. In a few isolated weekly intervals, feed consumption was statistically significantly lower (by a maximum of -1.13-fold) among rats administered the experimental phytase than those administered the control vehicle (Table 2). However, these occurrences were few, sporadic, detected in males only and not dose related. These differences were therefore considered unrelated to the test article.

**Table 2:** Feed consumption (g/animal/day) for male and female rats administered a daily dose of an experimental phytase by oral gavage for 90 days.

Treatment group		Experimental test period (days)												
		1−8	9−15	16−22	23−29	30−36	37−43	44−50	51−57	58−64	65−71	72−78	79−85	86−90
*Males*														
Control	Mean	30.9	34.0	34.4	35.7	36.0	36.4	36.1	35.9	36.2	35.0	34.9	35.5	32.5
	SD	1.67	2.48	2.75	2.90	3.14	3.28	3.22	2.85	3.53	2.85	3.06	2.86	3.15
	N=	10	10	10	10	10	10	10	10	10	10	10	10	10

T1	Mean	30.6	33.1	34.0	34.3	34.0	34.3	33.3	34.3	33.5	33.1	32.9	32.1*	31.5
	SD	1.98	1.83	2.59	2.65	2.53	2.46	2.90	2.28	1.74	2.05	1.84	2.30	2.39
	N=	10	10	10	10	10	10	10	10	10	10	10	10	9

T2	Mean	30.0	33.1	32.5	33.1	32.2*	32.2*	32.2	33.1	32.5*	32.4	31.5*	31.3**	30.4
	SD	0.81	0.82	1.58	1.11	0.81	0.89	0.55	1.12	1.21	0.95	1.32	1.57	1.79
	N=	10	10	10	10	10	10	10	10	10	10	10	10	10

T3	Mean	31.8	33.9	36.4	36.6	36.0	36.5	36.2	36.7	35.7	35.1	34.2	34.5	32.6
	SD	0.95	2.42	3.02	3.31	2.95	2.60	3.67	2.95	2.87	2.98	2.77	2.63	3.51
	N=	10	10	10	10	10	10	10	10	10	10	10	10	10

*Females*														
Control	Mean	19.5	19.9	22.1	22.0	21.4	21.6	21.0	21.45	21.1	19.9	19.9	20.4	18.8
	SD	0.94	1.11	2.51	1.52	1.35	1.28	1.63	1.45	1.92	1.31	1.38	1.60	1.37
	N=	10	10	10	10	10	10	10	10	10	10	10	10	10

T1	Mean	19.6	20.8	21.8	22.2	21.9	21.9	21.2	21.5	22.0	21.6	21.4	21.3	21.2
	SD	2.63	3.40	3.09	2.98	3.29	3.14	3.12	4.58	3.80	3.02	2.65	2.81	3.53
	N=	10	10	10	10	10	10	10	10	10	10	10	10	10

T2	Mean	19.9	20.4	21.2	21.1	21.1	21.4	20.9	21.4	20.9	20.5	20.7	20.0	19.7
	SD	1.21	0.89	1.23	1.26	1.15	1.15	1.77	2.29	1.17	1.38	1.56	1.82	1.78
	N=	10	10	10	10	10	10	10	10	10	10	10	10	10

T3	Mean	19.0	20.7	21.2	21.0	19.9	20.1	19.8	19.7	20.0	19.4	19.1	19.0	18.1
	SD	0.74	2.66	1.79	1.04	0.82	1.05	0.71	0.81	0.79	1.09	0.83	1.02	1.10
	N=	10	10	10	10	10	10	10	10	10	10	10	10	10

Control = distilled water.

SD = standard deviation.

T1 = experimental phytase at 250 mg TOS/kg bw/day (equivalent to 112,500 FTU/kg bw/day).

T2 = experimental phytase at 500 mg TOS/kg bw/day (equivalent to 225,000 FTU/kg bw/day).

T3 = experimental phytase at 1000 mg TOS/kg bw/day (equivalent to 450,000 FTU bw/kg/day).

* significantly different from control, at *P* < 0.05.

** significantly different from control, at *P* < 0.01.

**Table 3:** Relative organ weights (as a percentage of total final body weight) of male and female rats administered a daily dose of PhyG by oral gavage for 90 days.

	Treatment group											
Parameter	Control			T1			T2			T3		
	Mean	SD	(n)	Mean	SD	(n)	Mean	SD	(n)	Mean	SD	(n)
*Males*												
Final body weight (g)	615.1	67.91	10	579.8	62.48	10	576.0	48.71	10	621.8	79.79	10
Adrenal glands (% bw)	0.0109	0.0015	10	0.0122	0.0018	9	0.0109	0.0018	10	0.0110	0.0018	10
Brain (% bw)	0.3675	0.0339	10	0.3954	0.0413	9	0.3863	0.0317	10	0.3609	0.0381	10
Epididymis (% bw)	0.2426	0.0340	10	0.2726	0.0431	9	0.2594	0.0254	10	0.2412	0.0260	10
Heart (% bw)	0.3021	0.0178	10	0.3023	0.0168	9	0.3233	0.0335	10	0.3115	0.0213	10
Kidneys (% bw)	0.7044	0.0569	10	0.7356	0.0524	9	0.7095	0.0609	10	0.7217	0.0562	10
Liver (% bw)	2.9031	0.2362	10	3.2559	1.1733	9	2.8472	0.1669	10	3.2458	0.5826	10
Lung with bronchi (% bw)	0.3023	0.0227	10	0.3305	0.0453	9	0.3140	0.0260	10	0.2996	0.0358	10
Pituitary gland (% bw)	0.0023	0.0005	10	0.0026	0.0003	9	0.0027	0.0005	10	0.0025	0.0003	10
Prostate and seminal vesicles with coagulating gland (% bw)	0.4360	0.0733	10	0.4733	0.0612	9	0.4572	0.0732	10	0.4090	0.0665	10
Salivary glands (% bw)	0.1262	0.0207	10	0.1501**	0.0134	9	0.1296	0.0152	10	0.1268	0.0135	10
Spleen (% bw)	0.1420	0.0150	10	0.1918	0.1022	9	0.1503	0.0204	10	0.1587	0.0214	10
Testes (% bw)	0.5871	0.0929	10	0.6470	0.0845	9	0.6420	0.0750	10	0.6084	0.0795	10
Thymus (% bw)	0.0752	0.0196	10	0.0737	0.0092	9	0.0775	0.0138	10	0.0700	0.0161	10
Thyroid and parathyroid glands (% bw)	0.0054	0.0010	10	0.0046	0.0006	9	0.0050	0.0006	10	0.0047	0.0007	10

*Females*												
Final body weight (g)	288.0	20.34	10	301.9	64.82	10	294.5	29.62	10	276.5	18.79	10
Adrenal glands (% bw)	0.0298	0.0050	10	0.0284	0.0054	10	0.0264	0.0027	10	0.0293	0.0050	10
Brain (% bw)	0.7157	0.0520	10	0.6988	0.0927	10	0.7006	0.0681	10	0.7347	0.0558	10
Heart (% bw)	0.3732	0.0294	10	0.3574	0.0461	10	0.3511	0.0171	10	0.3633	0.0198	10
Kidneys (% bw)	0.7746	0.1082	10	0.7427	0.0580	10	0.7256	0.0613	10	0.7649	0.0559	10
Liver (% bw)	2.9011	0.2497	10	2.8462	0.2713	10	2.7797	0.3057	10	2.9099	0.2361	10
Lung with bronchi (% bw)	0.4752	0.0405	10	0.4832	0.0845	10	0.4679	0.0345	10	0.4891	0.0476	10
Ovaries (% bw)	0.0285	0.0057	10	0.0319	0.0070	10	0.0321	0.0080	10	0.0316	0.0048	10
Pituitary gland (% bw)	0.0068	0.0020	10	0.0062	0.0018	10	0.0068	0.0011	10	0.0068	0.0018	10
Salivary glands (% bw)	0.1589	0.0158	10	0.1588	0.0274	10	0.1576	0.0185	10	0.1586	0.0192	10
Spleen (% bw)	0.1860	0.0355	10	0.1785	0.0294	10	0.1833	0.0330	10	0.1785	0.0228	10
Thymus (% bw)	0.1087	0.0295	10	0.1025	0.0140	10	0.0968	0.0170	10	0.1059	0.0160	10
Thyroid and parathyroid glands (% bw)	0.0071	0.0010	10	0.0060	0.0009	10	0.0062	0.0015	10	0.0074	0.0018	10
Uterus/cervix (% bw)	0.2926	0.1609	10	0.2422	0.0826	10	0.2803	0.0965	10	0.2637	0.0856	10

SD = Standard deviation.

T1 = experimental phytase at 250 mg TOS/kg bw/day (equivalent to 112,500 FTU/kg bw/day).

T2 = experimental phytase at 500 mg TOS/kg bw/day (equivalent to 225,000 FTU/kg bw/day).

T3 = experimental phytase at 1000 mg TOS/kg bw/day (equivalent to 450,000 FTU/kg bw/day).

** significantly different from control, at *P* < 0.01.

#### 3.1.5 Neurobehavioral evaluation

There were no adverse or test article related changes in abbreviated neurobehavioral evaluations in male or female rats.

#### 3.1.6 Hematology and coagulation

There were no treatment-related changes in hematology parameters, red blood cell morphology or clotting potential as determined by analysis of prothrombin time, in male or female rats.

#### 3.1.7 Clinical chemistry

There were no treatment-related changes in clinical chemistry parameters in male or female rats.

#### 3.1.8 Urinalysis

There were no statistically significant differences in any of the measured urinalysis parameters among treatment groups, in male or female rats.

#### 3.1.9 Hormone analysis

There were no statistically significant differences in hormone analysis parameters among treatment groups, in male or female rats.

#### 3.1.10 Organ weights

No differences in absolute organ weights or in organ weights relative to brain weight were found among treatment groups, in male or female rats. When expressed as a percentage of bw the only significant difference was found for salivary glands of male rats who received the test article at 250 mg TOS/kg bw/day were heavier than those of male rats in the control group (1.19-fold increase; Table 3). However, this effect was not observed in female rats, and there were no correlated microscopic findings or effects in male rats at other dose-levels. The effect was therefore considered not test-article related. No other differences in organ weights relative to body weights in male or female rats were observed (Table 3).

#### 3.1.11 Gross pathology and histopathology

No treatment-related differences in gross pathology or histopathology of male or female rats were observed. All macroscopic and microscopic findings noted in males and females were considered incidental or spontaneous changes in rats of this species and age and were within normal ranges of severity and incidence. In one male rat (250 mg TOS/kg bw/day treatment group), malignant lymphoma was observed in the liver and spleen. This was considered a spontaneous neoplasm.

### 3.2 Genotoxicity studies

#### 3.2.1 Bacterial reverse mutation test (Ames test)

In both the initial toxicity-mutation assay and the confirmatory mutagenicity, no positive mutagenic responses were observed with any of the tester strains in either the presence or absence of S9 activation, under the test conditions. Neither precipitate nor toxicity was observed.

#### 3.2.2 *In vitro* mammalian chromosome aberration test

In both the preliminary toxicity assay and the chromosomal aberration assay, the test article was soluble in the treatment medium at all doses tested at the beginning and the end of the treatment period. The osmolality of the test article at the highest dose level (5000 μg/mL) was considered acceptable (<120 % of vehicle) and the pH at this dose level was 7.5. Cytotoxicity (≥ 50 % reduction in mitotic index relative to the vehicle control) was not observed at any dose in any of the three exposure groups in either assay. In the chromosomal aberration assay, no significant or dose-dependent increases in structural or numerical aberrations were observed at any dose in treatment groups with or without metabolic activity (p > 0.05 in all cases). All positive and vehicle control values were within acceptable ranges.

### 3.3 Similarity to protein toxins

The BLAST search against the complete Uniprot database revealed 427 matches based on an *E*-value cut-off of 0.1. Matches were to other phytases, phosphatases, and periplasmic proteins.

## 4 Discussion

Advancements in science and technology over the last 30 years have facilitated the production and development of increasingly more efficacious phytases. Recombinant DNA technologies have enabled the use of host microbial organisms as vehicles (‘production strains’) for producing large quantities of phytases identified as having desirable characteristics, by insertion of the candidate phytase gene into the host genome followed by expression using an appropriate promoter system [31]. Thus, the safety of both the enzyme itself and the production strain must be considered when assessing new phytases for commercial applications.

*T. reesei* was used as the microbial production strain in the present study. *T. reesei* has been widely used for the industrial manufacture of enzymes as feed additives (and for other applications) because of its capacity to produce extracellular enzymes rapidly and in large quantities [32]. The species has a long history of safe use in the biotechnology industry [33]. It is a non-pathogenic fungus and does not produce antibiotics or mycotoxins under commercial enzyme production conditions. It has been found suitable by the US EPA for exemption from full notification procedures required by the Biotechnology rule under the Toxic Substances Control Act (TSCA) for new recipient microorganisms being manufactured for commercial use, claiming this use would not present an unreasonable risk to injury to health or the environment [34]. The EPA has recently announced its intention to publish a formal regulation exempting *T. reesei*. Further, *T. reesei* has been classified as a Biosafety Level 1 microorganism by the ATCC meaning that it is not known to cause disease in healthy adult humans and has been listed as a safe production organism for industrial enzyme production in three major reviews on the safety of microbial enzymes [25,35,36].

The conduct of repeated toxicological testing with enzymes derived from the same strain lineage (*e.g*., *Trichoderma reesei*) with no adverse effects allows for the establishment of a Safe Strain Lineage (SSL) [26,[35], [36], [37]]. A SSL can be established by repeated assessment of members of the lineage using the Pariza and Johnson [35] decision tree, which involves addressing the pathogenic potential and potential for antimicrobial activity and toxic microbial metabolites and includes a repeat dose 90-day oral toxicity study to produce a No Observed Adverse Effect Level (NOAEL) to determine the margin of safety (MOS) in the intended use. Once the SSL has been established, the decision tree allows for other strains within the lineage that have undergone the same mutagenesis steps to be supported as safe production hosts for other enzymes, and any new enzyme preparations from the new strains in the lineage would also be considered safe. If a new branch of the lineage is developed based on random mutagenesis, then it would be advised to re-establish the safety of that new branch with toxin analysis, whole genome sequencing, and preferably, toxicology studies. This approach has been accepted, case-by-case, by the US FDA, several international agencies, and was recently adopted by JECFA in a recent proposal to update the JECFA food enzyme evaluation guidelines (https://www.who.int/docs/default-source/food-safety/final-enzyme-report-1-3-f-draft-nov-2019e4cf41981c82443bb2c5496a9adda8c4.pdf?sfvrsn=8651b8a5_4). The specific recombinant *T. reesei* strain used in the present study is part of a wider lineage of mutant *T. reesei* strains that has been developed by DuPont Nutrition and Biosciences through a combination of classical mutagenesis, selection and genetic engineering techniques from an original wild type parent strain (known as QM6a). Studies of the pathogenic/toxic potential of other strains within this lineage and of the recombinant enzymes produced by those strains (including *in vivo* oral toxicity testing and *in vitro* genotoxicity testing) have not indicated any safety concerns as reported in numerous GRAS Notices to FDA (*e.g.*, GRN 230, 315, 333, 372, 567, 703, 727, and 808). The current set of toxicology studies confirms the safety of the new branch of this lineage per suggestion by Ladics and Sewalt [26] and Ladics et al. [38].

Enzymes themselves are generally considered as safe *via* oral exposure due to their inherent presence in human and animal food, their digestion in the gastrointestinal tract and their natural production and presence in the gastric juices of animals [25,26]. Evidence suggests that microbial enzymes do not produce acute or subchronic toxicity and are not genotoxic. The results of the mutagenicity and chromosomal aberrations assays performed on this novel phytase are consistent with this, confirming that this phytase produced in a safe production organism bears no relevance to potential genotoxicity. PhyG did not cause a positive mutagenic response with any of the tester strains in either the presence or absence of metabolic activation, and a negative response was produced from the *in vitro* mammalian chromosomal aberration assay in HPBLs under the test conditions.

The bioinformatics analysis of potential toxin homology of PhyG indicated that the variant of a consensus bacterial phytase enzyme did not share significant homology with known protein toxins. Matches were to phosphoanhydride phosphorylases (phytases), phosphatases, and periplasmic proteins.

For the subchronic 90-day repeated dose oral toxicity study, PhyG was produced using a manufacturing process that was representative of the process used commercially. No evidence of *in vivo* toxicity was found in males or females when PhyG was administered at dose-levels of up to 1000 mg TOS/kg bw/day (equivalent to 450,000 FTU/kg bw/day). Small reductions (-1.13-fold maximum *vs*. control) in feed consumption observed in male rats fed the PhyG at 500 TOS mg/kg bw/day during several weekly time-points were not evident in females or in either sex at the higher dose-level, suggesting these effects were unrelated to the test article. Similarly, a higher (+1.19-fold *vs*. control) mean weight of salivary glands relative-to-body weight of male rats at the end of the study wasn’t test article related because the effect wasn’t 1) dose-related, 2) evident when absolute organ weights were compared and 3) evident in females. Malignant lymphoma observed in one male rat in the low-dose (250 mg TOS/kg bw/day) phytase treatment was unexpected but has previously been documented to be a common feature in multiple organs among older rats and sporadically reported in control males as young as 19 weeks old [39,40].

The NOAEL of 1000 mg TOS/kg bw/day from this subchronic toxicity study can be combined with information on the intended use level of the enzyme in the diet and with feed consumption data to generate a safety margin for the use of the enzyme in poultry/swine feed. Estimated daily feed intakes of broilers (scaled to body weight) are higher than those of pigs: default values of average daily feed intake for broilers, piglets and pigs for fattening, as determined by EFSA, are 79, 44 and 37 g DM/kg bw, respectively [41], and maximum daily feed intakes of poultry and pigs as determined by the NRC are 65 and 45 g DM/kg bw, respectively [42]. The present calculation is based on the EFSA value for broiler feed intake, this being the highest of all these values and therefore the most conservative. When PhyG is added to broiler feed, the likely recommended maximum inclusion rate will be 4000 FTU/kg feed. Based on this, the stated EFSA estimated daily feed intake for broilers, and an average feed dry matter content of 88 % [41], it can be estimated that an individual animal could be exposed to a maximum of 278 FTU/kg bw/day. This is equivalent to 0.62 mg TOS/kg bw/day (based on the calculated activity of the enzyme of 450 FTU/mg TOS; see section 2.1). To calculate the margin of safety it is convention to divide the NOAEL by the highest estimated exposure level, *i.e.* 1000 mg TOS/kg bw/day ÷ 0.62 mg TOS/kg bw/day = 1613. A margin of safety value of 100 or higher [*e.g.*, 200 for EFSA [43]] is generally considered as protective for human health, as demonstrated by the 100-fold safety factor applied to food ingredients by the US FDA (21 CFR 170.22). On this basis, the calculated margin of safety value of 1613 is indicative of an acceptable margin of safety for the test article when used in animal feed at a maximum recommended dose-level of 4000 FTU/kg feed.

## 5 Conclusion

The results of this study have demonstrated that a novel consensus bacterial phytase, PhyG, was not associated with any adverse effects in Crl:CD(SD) rats at dose-levels up to 1000 mg TOS/kg bw/day in a 90-day repeated dose subchronic oral toxicity study conducted in accordance with current OECD guidelines. *In vitro* genotoxicity testing and *in silico* protein toxin evaluation additionally confirmed PhyG to be non-genotoxic and not likely to pose a protein toxin risk upon consumption. A safety margin of 1613 was based on the established NOAEL, together with an estimate of broiler consumption and assuming incorporation of the phytase into animal feed at the maximum recommended level of 4000 FTU/kg. These findings support the safety of PhyG for the intended use as an animal feed additive.

## Declaration of Competing Interest

The authors declare that they have no known competing financial interests or personal relationships that could have appeared to influence the work reported in this paper.
